# Targeting the IGF-1R signaling and mechanisms for epigenetic gene silencing in human multiple myeloma

**DOI:** 10.3109/03009734.2012.659293

**Published:** 2012-04-19

**Authors:** Helena Jernberg-Wiklund, Kenneth Nilsson

**Affiliations:** Department of Immunology, Genetics and Pathology, Rudbeck Laboratory, Uppsala University, SE-751 85 Uppsala

**Keywords:** Epigenetics, IGF-1, multiple myeloma

## Abstract

Multiple myeloma (MM) is a B cell malignancy characterized by the expansion of clonal plasmablast/plasma cells within the bone-marrow. It is well established that the bone-marrow microenvironment has a pivotal role in providing critical cytokines and cell–cell interactions to support the growth and survival of the MM tumor clone. The pathogenesis of MM is, however, only fragmentarily understood. Detailed genomic analysis reveals a heterogeneous and complex pattern of structural and numerical chromosomal aberrations. In this review we will discuss some of the recent results on the functional role and potential clinical use of the IGF-1R, one of the major mediators of growth and survival for MM. We will also describe some of our results on epigenetic gene silencing in MM, as it may indeed constitute a novel basis for the understanding of tumor initiation and maintenance in MM and thus may change the current view on treatment strategies for MM.

## Introduction

Among the hallmarks of multiple myeloma (MM) is the characteristic accumulation of malignant cells with plasmablast/plasma cell phenotype preferably in the bone-marrow compartment. In many instances MM is preceded by monoclonal gammopathy of undetermined significance (MGUS), a pre-malignant state without clinical symptoms of malignancy (1). Unlike normal plasma cells, MM cells proliferate, although at a low rate (typically less than 1% of the cells display DNA synthesis at early disease stages), and have the capacity of prolonged survival and extended self-renewal. However, the mechanisms underlying such deregulation of the proliferation and differentiation processes as well as the apoptotic machinery in MM cells are only fragmentarily understood. Characteristic of the complex pathogenesis of MM is the genetic instability, as reflected by an extensive clonal variation between and within the tumor clones as displayed in patients and transgenic mouse tumor models (2-6). So far, a common pattern of structural and numeric chromosomal alterations has not been found, but genetic subgroups have indeed been identified (3). Although these have been used for sub-classification of MM, accumulating evidence shows that these identified genetic alterations are likely insufficient for malignant transformation (7). There is therefore still an urgent need to identify new genetic lesions, functional as common drivers of proliferation and stemness, markers for tumor progression, and, most importantly, rational biological targets for treatment in order to improve the outcome for patients with MM.

Highlighting the importance of the microenvironment for the initiation and maintenance of MM, the multi-faceted roles of insulin-like growth factor (IGF)-1 is now emerging, not only as a locally produced survival and growth factor, but also as a protein with novel functions, i.e. regulating gene expression, directly by shuttling to the nucleus (8), or indirectly, altering gene activation by histone modifications (9). Taken together, these novel findings further validate the benefits of targeting the IGF-1R in designed combinations, now also taking the epigenetic lesions into account, for therapeutic use in MM. By changing the focus from chromosomal aberrations to the use of an integrative genomics strategy, taking into account genetic as well as epigenetic alterations underlying a global RNA expression pattern in MM, we have recently contributed to the understanding of tumor initiation and maintenance of self-renewal in this plasma cell tumor (10).

## The role of genetic alterations in MM

Genetic aberrations in MM display a heterogeneous pattern with large intra- and interclonal variability. Most prominent of the genetic lesions are translocations to the Ig locus of five selected partner genes: 11q13 (cyclin D1), 6p21 (cyclin D3), 4p16 (fibroblast growth factor receptor 3 (FGFR3) and multiple myeloma SET domain (MMSET)), 16q23 (c-maf), and 20q11 (mafB) (11). MM is often, if not always, preceded by MGUS. However, not all cases of MGUS develop into overt MM. The genetic events mediating the transformation from MGUS to MM and the *de novo* transformation of MM are, however, largely unknown. Although IgH translocations are sometimes referred to as primary genetic events in MM transformation, they are also present in about 50% of MGUS (12,13). Therefore, these events are considered insufficient for malignant transformation. Cyclin D1 has been found to be uniformly and aberrantly activated by all these translocations in MM and has therefore been suggested to be a common downstream denominator of the transformation process. This finding, together with the fact that cyclin D is not expressed in normal hematopoietic cells and normal plasma cells, points to the possibility that there may be therapeutic windows for all molecular subtypes of MM by targeting this pathway (11). Although the prevalence of IgH translocations is increasing with disease stage of MM and present in >90% of human MM cell lines, their role in transformation of MM has been questioned by the fact that they are insufficient for recapitulating the process of initiating MM in transgenic mouse models (7). However, by refining the molecular classification based on the presence of IgH translocations and cyclin D gene expression, seven subclasses of MM are distinguishable (14,15). Considerable controversy, however, still exists concerning the clinical value of genetic lesions including IgH translocations as independent prognostic markers or valid as possible therapeutic targets (16,17). Secondary genetic events are numerous in MM, e.g. activating mutations of *K-* or *N-Ras* (18), disruption of the Rb pathway by inactivation/biallelic loss of Rb or p18INK (19,20), and PTEN mutations (21). The p53 status in subsets of MM is gaining new interest in molecular diagnostics, as p53 mutations were recently found to be tightly associated with monoallelic loss of 17p in poor-prognosis patients (22). Although aberrant expression of c-*myc* has usually been considered a late event in MM pathogenesis (23), c-*myc* was recently found to be activated during the transition from MGUS to MM in two-thirds of myeloma (24). This suggests that activation of c-*myc* may indeed occur early in the development of MM. Most likely multiple mechanisms, IgH translocations excluded, are involved in activation of c-*myc* (23). Pointing to one of these in the study by Chng et al. (24), the *myc*-induced gene signature corroborated, with few exceptions, with the presence of *ras* mutations in MM. In line with this regained importance of *myc* in MM pathogenesis, the interferon regulatory factor (IRF) 4, a direct target of c-*myc* activation, was recently identified among other oncogenic candidates to be indispensable for MM tumor growth, although sparsely involved in genetic alterations and translocations (25). Interestingly, mouse models displaying plasma cell tumors have recently been generated by conditional c-*myc* activation in GC B cells through the use of the physiological process of somatic hypermutation (SHM) (4). These alterations largely differ from the genetic alterations of *myc*, mainly translocations emerging in the classical mineral-oil-induced plasmacytoma initially described by Potter et al. (26). In contrast to this model, the conditional activation of *myc* in GC cells undergoing SHM in the Vk*myc model gave rise to tumors of postgerminal center origin (4). These tumors were indeed found to have biological and clinical features resembling human MM. The unique profile of *myc*-activated genes in MM, as compared to MGUS, suggests that *myc* during the transformation process may still represent a secondary genetic lesion in cells already hit by a primary event, i.e. IgH translocations or cyclin D1 activation (4).

Perhaps more powerful techniques, including high-throughput RNA-based profiling, taking into account copy number alterations, translocations, and epigenetic silencing by histone and DNA-based modifications, should be more useful in revealing crucial genetic transforming events, markers for poor prognosis, and common targets for therapeutic use. Recently, two large studies using these techniques report two independent sets of genes for the identification of poor-risk patient populations (27,28). Also, the first study using massively parallel sequencing of 38 MM genomes could confirm previously described mutations in MM, but it also identified novel genetic lesions, i.e. BRAF mutations, in a small fraction of MM patients that may indeed have implications for therapy (29). Successful strategies to connect expression profiles to specific signaling pathways have led to the discovery of activating mutations in the NF-κB pathway that certainly will prove important for new treatment strategies (30,31). Adding to this notion, the possibility of gaining knowledge of the mechanisms of gene activation in the tumor clone, i.e. by analysis of histone modifications of chromatin regulating gene silencing and constituting a basis for self-renewal and proliferation, was recently suggested by us (10). Taken together these findings may indeed prove gene profiles to be useful for prognostic purposes, for defining the nature of the tumor-initiating clone in MM, and for finding novel treatment strategies.

## The role of the microenvironment in supporting growth and survival of MM cells

It has become increasingly evident that the design of selective targeting drugs for tumor therapy should take into account also the survival benefits of the tumor–stroma interactions. It is an established fact that cell–cell contacts, cell–extracellular matrix interactions, and cytokines produced by the bone-marrow stromal cells are all important factors regulating growth and survival of MM cells (5,32–34). Several of these cytokines have been demonstrated to promote the growth, survival, and apoptosis of MM cells. Among these, IL-6 and IGF-1 were demonstrated by us and others to be major paracrine and autocrine growth and survival factors in MM cells *in vitro* and *in vivo* (34–38). It has also become increasingly clear that soluble factors may not only directly stimulate the expansion of the tumor clone, e.g. BAFF, APRIL, IL-10, and IL-15 (39), but in some cases (bFGF, VEGF) they may also indirectly contribute to tumor growth by triggering angiogenesis and/or production of growth-promoting factors by the surrounding normal bone-marrow cells, i.e. osteoblasts, fibroblasts, and endothelial cells (40,41). The prime focus of our research interest has in recent years been the IGF-1, a cytokine not only providing survival and growth benefits to the malignant plasma cells by autocrine stimulation and by paracrine signaling from the surrounding tumor stroma (38), but also by its function as a chemoattractant of MM cells guiding the tumor cells to the preferable site of growth in the bone-marrow compartment (42).

**Figure 1.**
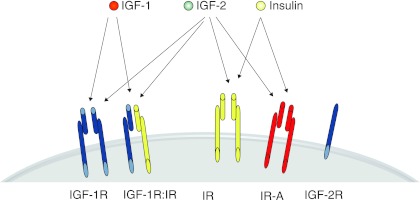
The most prominent ligand-receptor interactions with superior affinity over other ligands, i.e. IGF-1 to the IGF-1R and hybrids with either IR-A or IR-B, IGF-2 binds with high affinity IGF-1R, IR-A, and heterodimers with this isoform, while insulin displays high affinity for the IR and splice variants IR-A and IR-B only.

The complex IGF system consists of three ligands, IGF-1, insulin, and IGF-2, at least five cell-membrane receptors, IGF-1 receptor (IGF-1R), insulin receptor (IR), splice variants IR-A and IR-B, and, without obvious transmembrane signaling activity, the IGF-2 receptor (IGF-2R) (Figure 1). The extracellular α-subunit contains the ligand-binding domain of the IGF-1R, with affinity for IGF-1 and IGF-2 (43). Ligand binding induces a conformational shift in the covalently bound β-subunit now promoting attraction of adaptor proteins IRS, SHC, and Grb2 to the phosphorylated sites of the receptor. In turn, the SH2 domain containing proteins may transduce the signal to downstream activation pathways, including the extracellular signal regulated kinase (ERK) and protein kinase B (PKB/AKT), for proliferation and survival control. The formation of hybrid receptors of the IGF-1R has also been shown to contribute to tumor growth as shown in several *in vitro* tumor models, including breast carcinoma cell lines (44). As the IR exists in two isoforms (IR-A and IR-B), the formation of receptor hybrids hybrid-R with either IR-A or IR-B largely affects the outcome in signaling and biological consequences (45). Hybrid-Rs containing IR-A are activated by IGF-1, IGF-2, and insulin, while the IGF-1R–IR-B hybrid-Rs preferably bind IGF-1 with high affinity over IGF-2, and insulin (45) (Figure 1).

Adding to this complexity is the tight control of bioavailability of the IGF-1 by six high-affinity IGF binding proteins increasing the ligand half-life. In the light of this knowledge, it was perhaps not an unexpected finding that the mere presence of IGF-1 in serum, or the level of IGF-1R on the surface of tumor cells, has not been found to be a useful biomarker for tumor incidence or progression (46–48). Despite ubiquitous expression of the IGF-1R in cell types originating both from the epithelial and mesenchymal compartment, reports on the modest use of this receptor in the control of normal cell growth in the adult individual indicate the use of this receptor as an attractive drug target amenable for tumor therapy. Indicating the importance of the IGF-1R in oncogenesis in general, the receptor seems indispensable for transformation (49), and its overexpression in murine models may even increase the incidence of tumor formation (50).

A pivotal role of IGF-1R in the pathogenesis of MM was originally reported by us (34) and confirmed by others (37,51). Our previous studies have shown that IGFs and IGF-1R signaling play critical roles in stimulation of proliferation, survival, and drug-resistance of MM (34,52). IGF-1R-antagonistic antibodies (αIR3) interfering with the IGF-1 autocrine loop or with the IGF-1 axis were later shown to have comparable effects in profoundly suppressing serum-induced survival and potentiate apoptosis induced by glucocorticoids and death receptors, i.e. Fas, in MM cells (52,53,21,54). In a recent publication, the role of IGF-1 as a mediator of survival was clarified by linking the inhibition of apoptosis to the silencing of important effector genes (9). One of the genes significantly and consistently down-regulated by IGF-1 stimulation in human and murine MM cells is the Bim (Bcl2like11) gene, a member of the BH3-only group of the Bcl-2 protein family, and a mediator of apoptosis in pathways utilizing the mitochondrial pathway. Functional studies proved the importance of Bim in drug-induced apoptosis of MM. The silencing of Bim by RNAi strategies rendered MM cell lines *in vitro* refractory to bortezomib, melphalan, and histone deacetylation (HDAC) inhibitors. Analysis of histone modifications at the Bim promoter, and its upstream transcriptional activator, revealed that IGF-1 treatment of MM resulted in an increase of repressive histone methylation marks (H3K9me2) and thus reduced transcription of Bim (9). This clearly establishes a novel role for IGF-1 in regulating gene expression by epigenetic silencing of target genes in MM.

In line with this newly ascribed function, the IGF-1R recently also joined the group of transmembrane receptors, among these the EGFR, that may undergo translocation to the nucleus (55). Previous publications even suggest nuclear EGFR to be a prognostic indicator for poor clinical outcome and a mediator of aggressiveness of tumor cells (56). Shuttling of the IGF-1R to the nucleus and subsequent chromatin binding is a ligand-dependent process requiring tyrosine kinase activity and sumoylation of the IGF-1R (8,57). Likely, the IGF-1 as well as IGF-2 binding to the IGF-1R may induce intracellular transport of the receptor. The biological role of nuclear IGF-1R is essentially unknown, but it seems to be associated to proliferative capacity rather than to expression levels *per se*. Interestingly, the nuclear IGF-1R was also shown to be co-localized to RNA polymerase II on chromatin. Nuclear IGF-1R may be detected in proliferative benign epithelial cells, in solid tumors of breast, lung, and prostate, and also in MM (55,57). Detailed analysis of the contribution of the nuclear IGF-1R to the activated gene profile of IGF-1-induced signaling will certainly prove to be important.

IGF-1R signaling has been established to mediate survival in authenticated MM cell lines (5) and CD138+ primary MM cells. Its importance has also been validated *in vivo* in the syngeneic mouse models 5T3MM and 5T2MM, well representative for human MM (58). This model originated from spontaneously arising murine MM in C57BL/KaLwRij mice and may be maintained by serial transplantation in syngeneic mice (59). The 5TMM tumor-bearing mice have the advantage of being immunocompetent. The 5TMM tumor also closely resembles the human MM with respect to genetic alterations, tumor location to the bone-marrow, induction of angiogenesis at the tumor site, and the presence of osteolytic lesions (60). Proving the importance of studying the benefits of survival factors in MM in a syngeneic environment, IGF-1 was in this model found to induce VEGF production and angiogenesis and was certainly proving important for tumor growth. Thus, such a tumor environment where IGF-1 may directly and indirectly stimulate tumor expansion seems optimal for validating the therapeutic potential of developed antagonists to the IGF-1R signal in MM (61).

## Clinical implications of targeting the IGF-1R in MM cells *in vitro* and in the 5TMM model *in vivo*

A few selective inhibitors to the IGF-1R (antagonistic antibodies) or its kinase activation domain (small synthetic RTK inhibitors) have been described, some of these tested also in early clinical trials for solid tumors and MM (62,63). The development of drugs for selective targeting the IGF-1R was for long severely hampered by the lack of unique domains in the kinase activation domain of the IGF-1R (84% homology between the IGF-1R and insulin R) and the redundancy of the ligands emitting growth signals as previously described.

Our publications, describing studies with the small molecular inhibitor of the cyclolignan family picropodophyllin (PPP) *in vitro*, showed that selective inhibition of the IGF/IGF-1R pathway by this drug can be achieved with a favorable therapeutic window in MM cell lines and primary cells (61,64,65). Importantly, inhibition of the IGF-1RTK with PPP was originally designed to be non-competitive with ATP, suggesting interference with the IGF-1R at the substrate level (66). Most importantly the PPP did not show an inhibitory activity of the insulin receptor (IR) even at submicromolar concentrations as shown in *in vitro* and *in vivo* kinase assays (64–66). The investigations of the molecular mechanisms revealed that the inhibition of the IGF-1R by PPP was associated with growth arrest, caspase-dependent and -independent apoptosis in human MM cells, and a reduced expression of anti-apoptotic genes including Mcl-1. However, not until proving its potential in a therapeutic setting could the IGF-1R be considered a potential therapeutic target in MM. Indeed, a successful eradication of the tumor clone by PPP in the therapeutic setting of the 5TMM *in vivo* model (61,64,65) set the stage for the use of inhibition of the IGF-1R in MM therapy. In 5T2MM mice, treatment with PPP also had favorable effects on the osteolytic process. The number of osteolytic lesions was almost completely blocked as a result of PPP treatment. Importantly, the non-toxic effect of long-term treatment in the mouse model was encouraging, with no cytotoxic effects or elevated serum glucose levels (61). An oral IGF-1R inhibitor (AXL-1717) of cyclolignan is currently in phase I/II in subjects with advanced cancer (www.axelar.se).

Results from preclinical studies using other strategies inhibiting the IGF-1/IGF-1R now show that some of the efforts to uniquely antagonize the receptor at various levels of its signaling pathway have been successful (63). Several of the humanized IGF-1R antibodies and small receptor tyrosine kinase (RTK) inhibitors have been subjected to investigations in MM in *in vitro* studies, preclinical mouse models of MM, and in clinical trials (51,67,68). So far, the results from the first clinical studies using antagonistic antibodies have unfortunately not lived up to the high expectations based on the results from the preclinical studies. Taking into consideration the severe cytotoxic side effects reported for antagonistic antibodies in phase III trials, small molecule IGF-1RTK inhibitors may be preferable to antagonistic antibodies for clinical use (63,69).

In our preclinical setting for PPP, using the *in vivo* MM mouse model 5TMM, the tumor in the treated mice eventually relapsed (61). The mechanisms behind the development of refractory tumors are currently under investigation. The important study by Hashemi et al. (70) may shed some light on underlying mechanisms leading to acquired insensitivity to PPP. The genetic alterations induced as a result of PPP treatment in cultured solid tumor cells *in vitro* are numerous. In a few cases the altered genes were shown and validated by siRNA experiments to be connected with IGF-1R function, i.e. *SOCS3*, *BCL2*, and *MAPK*. Importantly, only a reversible resistance developed in the presence of PPP, even after long-term cultivation of both solid tumors and MM *in vitro* (71) (Jernberg-Wiklund, unpublished observations). The reversible resistance did not include genetic alterations, e.g. amplifications of several common genes coding for resistance to drugs (70). Importantly, although not supported by the *in vitro* findings with respect to drug-induced resistance, the development of drug-resistant clones in the *in vivo* model should not be unexpected. The complex network of IGF-1R signaling is likely to create feedback signals which may call for combinatorial designs for improved treatment. Efficient blockade of the IGF-1R was previously suggested to induce a compensatory response *in vivo*, i.e. leading to an up-regulation of IGF-1 in serum (46). A recent example of compensatory signals from the complex IGF network is the IGF-2 ligand binding and signaling via insulin receptors (IR), contributing to the intrinsic resistance to anti-IGF-1R antibody treatment in mouse models of pancreatic tumors (72). However, using the small synthetic IGF-1R inhibitor PPP we could show that excess amounts of IGF-2 or insulin did not overcome the growth inhibitory effects *in vitro* (64). Considering the low levels of circulating IGF-2 in murine serum, a possible feedback signaling from the IGF-2 via IR receptors *in vivo* should not be an expected complication in this model. So far, encouraging data and absence of severe side effects reported from the early clinical trial of the AXL-1717 (73) indicate that feedback mechanisms as reported by Ulanet et al. (72) might be of less importance using small synthetic RTK inhibitors.

To counteract the problem of acquired drug resistance *in vivo*, our group and others have embarked on the development of combinatorial therapies for MM. We have focused predominantly on pairing promising novel anti-tumor agents with agents conventionally used in MM management. So far, one of the most successful achievements of apoptosis sensitization was found by combining IGF-1R or IGF-1R downstream components, i.e. mTOR inhibitors, with glucocorticoids (74). The selective inhibitor of mTOR, rapamycin, exhibiting only cytostatic effects when used as a single agent in MM models *in vitro*, mediated a significant potentiation of dexamethasone-induced apoptosis and suppressed constitutive, serum-, IGF-1-, and IL-6-induced survival in MM cell lines, in purified cells from MM patients (74). Our studies and those of others on the molecular mechanisms underlying the observed sensitization to apoptosis revealed an inhibition of both mTOR- and MEK/ERK-mediated phosphorylation of the kinase p70^S6K^ at required sites for activation, as well as down-regulation of the cell cycle-regulated proteins cyclin D2 and D3 (74,75). These studies provide a proof-of-principle that abrogating parallel survival factor pathways from the IGF-1R by use of rapamycin will render cells susceptible to drug-induced apoptosis, especially if the underlying mechanism of this combination is that the drug and inhibitor co-operate by blocking all activations sites on a common target protein, the p70^S6K^.

This approach points to the possibility that combinations of multiple investigational agents with drugs intervening with the IGF-1R pathway should be useful in MM. Several preclinical studies support the use of IGF-1/IGF-1R inhibitors in combination with other drug strategies. In MM the use of humanized anti-IGF-1R antibody IMC-A12 (ImClone) has been shown to act in synergy with mTOR inhibitors, bortezomib and melphalan, in MM models *in vitro* and *in vivo* (68,76). Enhanced effects have also been reported by use of ATP competitive IGF-1RTK inhibitors (Novartis; AEW541, ADW742) in combination with dexamethasone, lenalidomide, and bortezomib (46,77). The combination of mTOR inhibitors and IGF-1R inhibitors is currently under investigation in clinical studies (63).

In a recent study by our group the use of synergistic drug combinations with PPP has been thoroughly investigated by using high-throughput screening (HTS) of drug libraries. Several potent compounds have been identified, including dexamethasone, rapamycin, and p38 inhibitors (64). Among these, enhanced effects on apoptosis enhancement, cell cycle arrest, and reduced tumor load in the 5TMM model *in vivo* were achieved by combining a novel HDAC inhibitor, LBH589 (panobinostat), with the IGF-1R inhibitor PPP (78). The mechanisms underlying the synergy of this combination are currently under investigation.

## The role of epigenetic gene regulation by the Polycomb group proteins (PcG) in MM

Although MM cells phenotypically closely represent postgerminal center plasmablasts/mature plasma cells, the precise nature of the target cell of transforming mutations in MM is still unknown, as is the question of the existence and characteristics of tumor-initiating cells in MM possibly harboring self-renewal capacity.

**Figure 2.**
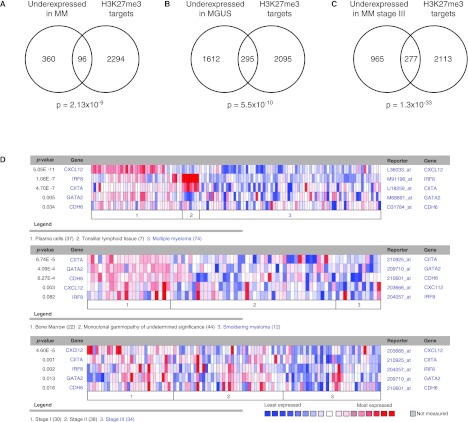
The H3K27me3 target genes defined by Bracken et al. (81) in embryonic fibroblasts are statistically associated to underexpressed genes in MM and MGUS using the data-mining platform Oncomine. A: The top 10% underexpressed genes in MM patients (compared to normal bone-marrow) show a strong connection to genes previously described as H3K27me3 targets in human embryonic fibroblasts. B: The H3K27me3 target genes were also overrepresented among genes underexpressed in MGUS patients, and (C) strongly associated with decreased expression in ISS stage III MM, compared to stage I and II. D: The PRC2 targets CIITA, GATA2, CDH6, CXCL12, and ICSBP/IRF8 are underexpressed in MM. From Kalushkova et al. (10) with permission.

Note: Colors are z-score normalized to depiet relative values within rows. They cannot be used to compare values between rows.

In our aim to dissect the nature of the tumor-initiating capacity in MM, we performed an integrative genomics approach based on the differences in gene expression between non-malignant and malignant plasma cells (Figure 2). By using this approach, we could define a silenced profile consisting of the top 10% most underexpressed genes in MM patients as compared to normal non-malignant plasma cells from bone-marrow and tonsillar tissue (79,80). The silenced genes had a common denominator in that they were histone H3 tri-methylated at lysine 27 (H3K27me3) and targeted by the Polycomb repressive complex 2 (PRC2) as previously defined by Bracken et al. (81). Interestingly, the underexpressed gene profile in MM showed a significant overlap to the well-described literature-based concept of H3K27me3 and EZH2 target genes linked to the self-renewal capacity of stem cells (82,83). Indicating the clinical relevance of this finding, the suppression of this gene profile was more pronounced in the advanced stages (ISS stage III as compared to stages II and I) of MM progression (Figure 2) (10).

The Polycomb repressive complexes 1 and 2 (PRC1 and PRC2) are multiprotein Polycomb group (PcG) complexes, among which the PRC2 is involved in the initiation of gene repression (84). The silencing of genes marked by the PcG proteins is an established part of the epigenetic memory during embryogenesis repressing lineage-specific developmental genes (85,86). The core PRC2 complex is formed by the suppressor of zeste 12 homolog (Drosophila) (SUZ12), the embryonic ectoderm development (EED), the RbAp46/48 (also known as RBBP7/4), and the enhancer of zeste homologs 1 and 2 (Drosophila) (EZH1/EZH2)—the catalytic subunits harboring the histone methyltransferase activity that mediates tri-methylation on H3K27 (H3K27me3) (84). Interestingly, to increase plasticity of the chromatin structure, tri-methylation of H3K27 may be part of a bivalent nucleosome mark occurring together with chromatin marks permissive for transcription, such as methylation of lysine 4 (H3K4me) (87). After the removal of the silencing H3K27me3 mark individual genes are considered ready for activation, allowing cells to differentiate to their allocated destination.

Our results are in agreement with several recent genome-wide studies implicating Polycomb-mediated gene silencing in the development of malignancy in solid tumors (83,88). In multiple types of lymphoma, breast and prostate cancer, the aberrant expression of EZH2 is also indicative of poor prognosis and metastatic tumor (85,89). Also, in MM the overexpression of EZH2 and other PcG proteins has been confirmed (10). Several possibilities exist to explain the constitutive expression of EZH2 in MM, including the aberrant expression of c-*myc* as mentioned previously (24). Thus it is possible that tumor initiation and progression in MM is not only driven by genetic alterations but also by epigenetic events that confer stemness to a proliferating cell clone devoid of terminal differentiation capacity. Our findings were recently supported by a report demonstrating another core constituent of the Polycomb-repressive complex 1, the Bmi-1, to be essential for the maintenance of the transformed phenotype of myeloma cells both *in vitro* and *in vivo* (90). In line with our findings it is certainly possible that Bmi-1 of the PRC1 maintains silencing originally initiated by the PRC2 complex.

Because primary bone-marrow MM cells have a very low clonogenic capacity in agarose *in vitro*, and a low tumor-forming potential in nude mice, the expansion of MM clones *in vivo* has been suggested to depend on a small population of tumor cells with self-renewal capacity, ‘MM tumor stem cells’ (91). Matsui et al. (92) showed that only CD138- MM cells had the capacity of forming tumors in NOD/SCID mice *in vivo*, while the bulk of inoculated CD138+ MM cells did not have this capacity. Recently, the view that CD138- MM cells represented a small population of ‘MM tumor stem cells’ has been questioned. Using the *in vivo* model of 5TMM, Van Valckenborgh et al. (93) have clearly demonstrated that both CD138+ and CD138- MM populations indeed have similar clonogenic capacity and are tumor-initiating *in vivo* (93). Interestingly, the CD138- MM cells were, however, less proliferative and displayed gene expression corroborating the idea of a less mature phenotype than the CD138+ MM cells. The identification of the Polycomb-silenced gene profile in MM patient samples and cell lines now also implies that this signature is a general feature shared by a large proportion of the MM cells rather than confined to a small subpopulation of ‘MM tumor stem cells’. Importantly, these findings may call for a novel concept of tumor stemness and proliferative capacity in MM.

**Figure 3.**
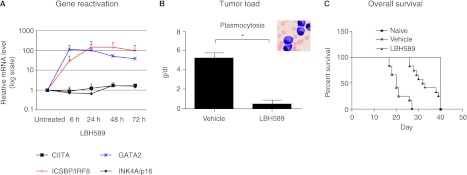
A: In a preclinical setting a few selected candidate target genes from the integrative genomic analysis were analyzed and found to be reactivated by chemical inhibitors in MM cell lines *in vitro*. B: Treatment with LBH589 significantly reduced tumor load in the 5T3MM mouse model, and also increased overall survival in this model (C). From Kalushkova et al. (10) with permission.

An emerging question is if targeting the common denominator for the observed gene silencing can be used in the treatment of MM, and, if so, whether this may have consequences as inhibited cell growth *in vitro* and *in vivo*. Although Polycomb target genes silenced by EZH2 seem more prone to be permanently silenced by DNA methylation, possibly as a consequence of direct recruitment of DNA methyltransferases DNMT1 and 3 to the PcG, recent studies in prostate cancer suggest that EZH2-mediated histone methylation and DNA methylation may indeed occur side by side in gene silencing (94,95). Thus epigenetic gene silencing may in fact be an attractive drug target due to the fact it can be reverted and in relevant cases combined with DNA demethylating drugs, i.e. 5-azacytidine. Initially in the paper by Kalushkova et al. (10) two chemical inhibitors, the global histone methylation inhibitor 3-deazaneplanocin (DZNep) and the histone deacetylase inhibitor LBH589, were used to show proof-of-principle that we may indeed successfully reactivate selected genes carrying the silencing H3K27me3 mark. DZNep and LBH589 (panobinostat) both reactivated the expression of selected genes repressed by H3K27me3, depleted cells from the PRC2 component EZH2, and induced apoptosis in human MM cell lines (Figure 3).

In the 5T33MM *in vivo* model for MM, treatment with LBH589 thus resulted in up-regulation of genes in the signature, reduced tumor load, and increased overall survival (Figure 3). The drugs used for proof-of-concept may certainly have effects other than targeting EZH2. However, no toxic side effects on normal blood cells and fibroblasts were monitored at concentrations inducing apoptosis in MM *in vitro* (10). Certainly, more drugs selectively targeting the components of the PRC2 and PRC1 complex are needed to unravel the therapeutic potential of reactivating the silenced gene signature in MM.

## Concluding remarks

A critical link between IGF-1R signaling and transformation makes this receptor an excellent target for inhibition of survival circuits in human tumors including MM. Adding to our original finding that IGF-1 is an important paracrine and autocrine growth and survival factor, recent reports suggest that IGF-1 may also regulate gene expression via epigenetic events and via nuclear transportation (8,9,38). Extensive preclinical studies have proved the benefits of small molecular compounds with selective activity to the IGF-1R (51,61,64,65), now in clinical trials with encouraging results so far (73).

In the design of novel drugs for MM, the current view on the presence of a small population of tumor-initiating cells uniquely harboring self-renewal capacity and decreased sensitivity or resistance to drugs should also be challenged. Although the CD138- MM cells might exhibit less proliferative potential, these cells apparently do not constitute the entire population of ‘MM tumor stem cells’. As proven by recent findings (93), also the CD138+ MM tumor cells share a similar capacity for clonogenic growth and tumor initiation *in vivo*. In line with this, we recently published an integrative genomic analysis, where tumor stemness and maintenance of proliferative capacity may be defined by a Polycomb-silenced gene profile in MM patient samples and cell lines (10). This profile is likely a general feature shared by a large proportion of MM cells rather than restricted to a small population of ‘MM tumor stem cells’. The target cell and the initial genetic events for transformation being unknown in MM, the mechanisms underlying the acquired Polycomb gene repression signature in late stages of plasma cell differentiation remain to be clarified.
